# Identification of *ALOX12B* Gene Expression, Evolution, and Potential Functional Sites in Horn Development of Sheep

**DOI:** 10.3390/ijms26010079

**Published:** 2024-12-25

**Authors:** Ran Lv, Guoqing Zhang, Hao Li, Jianxin Shi, Zhu Meng, Xiaoning Lu, Mingzhu Shan, Jie Yang, Zhangyuan Pan

**Affiliations:** 1 College of Life Science and Technology, Xinjiang University, Urumqi 830047, China; lvr1999@126.com; 2 State Key Laboratory of Animal Biotech Breeding, Institute of Animal Science, Chinese Academy of Agricultural Sciences (CAAS), Beijing 100193, China; 3 National Nanfan Research Institute (Sanya), Chinese Academy of Agricultural Sciences (CAAS), Sanya 572024, China

**Keywords:** *ALOX12B*, sheep horn, RNA-seq, ASE, WGS, SNPs

## Abstract

The growth and development of horns are primarily controlled by the skin. The *ALOX12B* gene is crucial for epidermal barrier function and may have a significant impact on horn growth. The purpose of this study was to investigate the expression of *ALOX12B* across different sheep breeds and tissues by utilizing RNA sequencing. Additionally, potential functional sites were identified in conjunction with whole genome sequencing. Our findings revealed that *ALOX12B* was highly expressed in the scurred horn group as opposed to the normal horn group (SHE). *ALOX12B* expression was also notably high in the skin across several species. Eight loci that may influence horn size were indicated in this study. Through the alignment of the ALOX12B protein sequence from 16 species, 15 amino acid sites were identified specifically expressed in horned animals. In conclusion, this study established a connection between *ALOX12B* and horn size and identified a series of functional sites that may serve as molecular markers for reducing the presence of horns in Chinese sheep breeds.

## 1. Introduction

Sheep hold significant importance as a livestock species in China, contributing significantly to food security and livestock products [1]. However, as animal husbandry advances, the presence of sheep horns increases feeding costs, poses risks to fencing facilities, and can harm both livestock and feeding personnel [2], leading to hidden dangers in sheep production management. Although members of the breeding industry believe that there is no direct correlation between the production performance of horned and hornless sheep, the trend has been towards the use of hornless breeds because of their ease of management [3]. The current artificial dehorning method creates animal welfare concerns, increases workload, causes physiological damage to individual sheep, and negatively impacts production performance. Therefore, genetic selection for hornless sheep has become a research priority [2].

Horns represent a crucial evolutionary feature in ruminants [4], playing an important role in self-defense, confronting predators, and competing for mating rights [5,6]. As derivatives of the skin, their growth and development are primarily regulated by the skin. However, from an economic value perspective, horns do not directly affect economic value features such as milk production, meat quality, or fur. Consequently, despite their evolutionary significance, horns are not considered an economically relevant trait in livestock production. Several genes connected to horn growth have been identified, such as *RXFP2*, *HOXD*, *NRDC*, *DCN*, *MYL2*, *INSL3*, *FOXL2*, *SFRP2*, *KRT1,* and *WNT3* [1,6,7,8,9,10]. However, the formation and regulatory mechanisms of sheep horns are still unclear. Our previous research on sheep horns assessed the gene expression variations between normal and scurred horn groups and discovered, through Gene Ontology (GO) enrichment analysis, that *ALOX12B* contributes to the skin development process.

The *ALOX12B* (arachidonate 12 lipoxygenase, 12R type) gene encodes the protein 12R-LOX, which is involved in fatty acid metabolism. 12R-LOX is mainly synthesized in the epithelial tissues (e.g., skin) and is essential to epidermal barrier function [11,12,13,14]. *ALOX12B* also exhibits immunosuppressive activity by reducing antigen presentation in T cells. Research on this gene has predominantly focused on humans, with very little research conducted on sheep. Genetic evidence has described this gene as the pathogenic gene of autosomal recessive congenital ichthyosis, which is a severe keratinization disease [11]. This indicated that *ALOX12B* had a significant role in skin keratinization [15]; its missense and nonsense mutations are widely present in various cancers, including skin, colorectal [15,16], and cervical [17]. However, no research has been conducted to document that *ALOX12B* has a relationship with horn size.

The present research aims to investigate the expression, evolution, and potential functional sites of *ALOX12B* by multi-omics analysis, to explore the molecular mechanisms of *ALOX12B,* and to provide valuable molecular markers for reducing the presence of horns in Chinese sheep breeds.

## 2. Results

### 2.1. Sample-Specific Expression of ALOX12B in Different Groups

**Figure 1:**
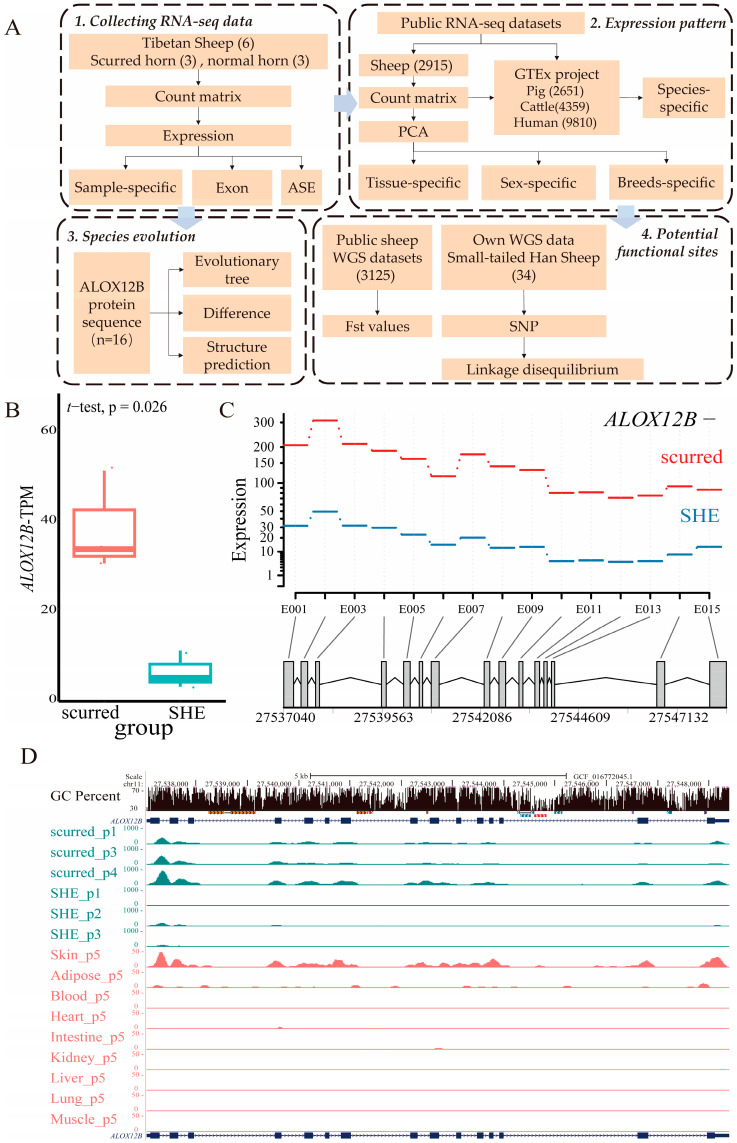
Sample-specific expression of *ALOX12B* in the normal (SHE, n = 3) and scurred horn group (n = 3). (**A**) Schematic illustration of the experimental procedure. (**B**) Comparison of *ALOX12B* expression levels between the SHE and scurred groups. The *p*-value was performed using a *t*-test. (**C**) Expression pattern of *ALOX12B* exons in the SHE and scurred horn groups. The expression values represent fitted estimates obtained by GLM regression, with the horizontal coordinate indicating exon numbers. Red denotes the scurred group, and blue indicates the SHE group. (**D**) Comparison of *ALOX12B* expression in soft horn tissues and other tissues. Green represents soft horn tissue, and red represents other tissues. The peak value represents the expression level of *ALOX12B*.

*ALOX12B*, located on chromosome 11, has a transcript: XM_004012678.5. This transcript was used for subsequent analysis. The experimental procedure is shown in Figure 1A. Using RNA sequencing (RNA-seq) data from self-tested Tibetan sheep (n = 6), this study indicated that the scurred horn group could express *ALOX12B* at a more elevated level (*p*-value = 0.026) (Figure 1B). This result showed that the scurred horn group (n = 3, horn lengths ranging from 0 to 12 cm) exhibited higher levels of exon expression than the normal horn group (SHE, n = 3, horn lengths > 12 cm) (Figure 1C). Further analysis of *ALOX12B* gene expression and GC percent was performed using the University of California Santa Cruz (UCSC) database (Figure 1D). Expression of all exons could be observed in the scurred horn group, whereas only minimal expression of Exon 1, Exon 2, Exon 4, and Exon 15 could be observed in the SHE group. Additionally, skin tissue exhibited a similar trend of exon expression as horn tissues but at much lower levels than those observed in horn tissues. In contrast to skin and horn tissues, these exons were largely not expressed in adipose, muscle, stomach, or kidney tissues.

### 2.2. Differential Expression of ALOX12B in Tissues, Species, Sex, and Breeds

In order to clarify the differential expression of *ALOX12B*, RNA-seq information of different humans (n = 9810), pigs (n = 2651), cattle (n = 4359), and sheep (n = 2915) tissues were collected from public databases, and we found expression differences of *ALOX12B* in 16 independent tissues. Overall, *ALOX12B* was significantly expressed in the skin of all species, with the highest TPM expression level in humans, followed by sheep. High expression of *ALOX12B* was also observed in the testis of both sheep and pigs. Sheep and pigs had similar expression levels.

**Figure 2:**
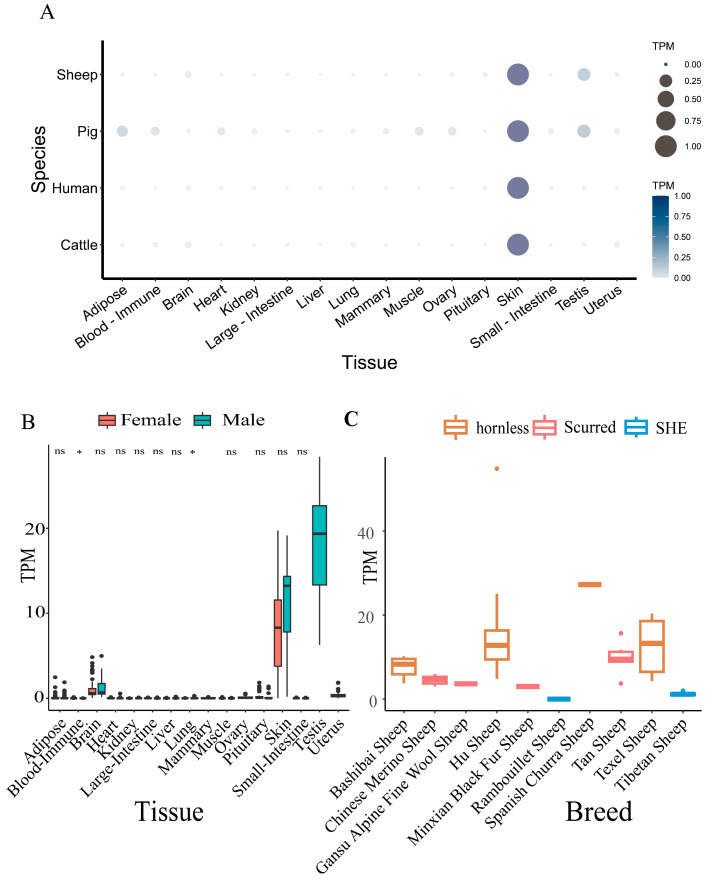
Differential variations in *ALOX12B* across species, tissues, sex, and breeds. (**A**) The expression levels of *ALOX12B* across different human (n = 9810), pig (n = 2651), cattle (n = 4359), and sheep tissues (n = 2915). The larger scatter and darker color mean a higher expression of *ALOX12B* in the tissues. (**B**) Sex-based differences of *ALOX12B* in different sheep tissues (n = 2915). Asterisks represent *p*-values from the *t*-test: * denotes a *p*-value < 0.05, and ns denotes a *p*-value > 0.05. (**C**) *ALOX12B* expression variations in skin tissue of different sheep breeds (n = 2915).

Additional research in the public data (n = 2915) was conducted to determine whether there were sex differences in *ALOX12B* (Figure 2B). High expression of *ALOX12B* was observed in the skin, with no significant sex differences found. At the same time, the expression level of *ALOX12B* was high in the testis. In the blood and lung tissues, although there were sex differences, its expression level was relatively low or almost nonexistent.

Additionally, the expression level of *ALOX12B* in skin tissue was compared among different sheep breeds (Figure 2C). These ten sheep breeds were divided into three groups (n = 2915): hornless, scurred, and SHE. The findings demonstrated that *ALOX12B* expression was highest in hornless sheep, then scurred sheep, and lowest in SHE sheep. As a scurred breed, Tan sheep had a slightly higher expression of the *ALOX12B* gene than the Bashibai sheep. These results indicated that the gene expression of hornless and scurred horn sheep was slightly different from each other.

### 2.3. Species Evolution and Structure Prediction of the ALOX12B Protein

**Figure 3:**
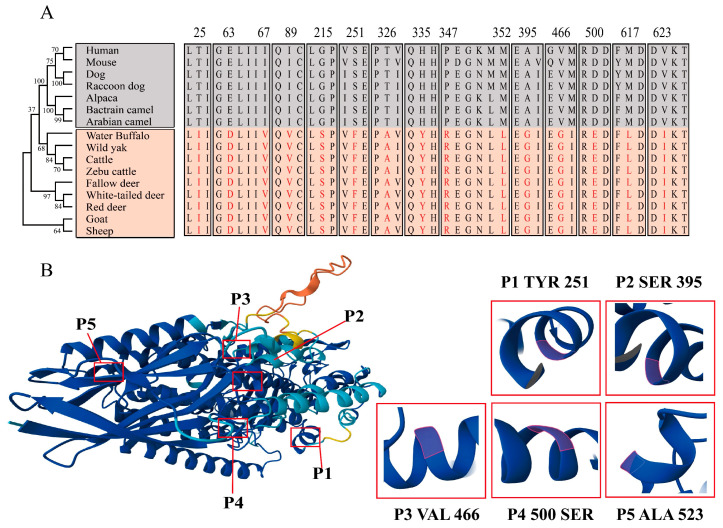
Evolutionary insights and structural prediction of the *ALOX12B* gene. (**A**) On the left is an evolutionary tree of the ALOX12B protein, alongside amino acids exclusive to horned animals on the right. Red highlights amino acids that differ between the two groups. (**B**) The three-dimensional structure of the sheep ALOX12B protein, predicted by AlphaFold2, with the lowest confidence in yellow and the highest in blue. A darker color means a higher confidence. On the right are five amino acids with an α-helix and their name, marked by a red box.

The phylogenetic relationships of 16 species are shown in Figure 3A. The ALOX12B protein evolution was found to align with the species evolution, with horned species such as cattle, deer, and sheep clustering together and hornless species such as humans, mice, dogs, and camels clustering together. The horned and hornless clusters were well separated, indicating that the structure of the ALOX12B protein is similar and that there has been some protein conservation in horned animals like cattle and deer. The 15 amino acids located at positions 25, 63, 67, 89, 215, 251, 326, 335, 347, 352, 395, 466, 500, 617, and 623 are distinctive to horned animals, which may be vital in determining whether sheep have horns. The amino acids 251, 395, 466, 500, and 623 were located in the α-helix with higher confidence. These positions could make a substantial impact on horns (Figure 3B).

### 2.4. Allele-Specific Expression Analysis of ALOX12B

**Figure 4:**
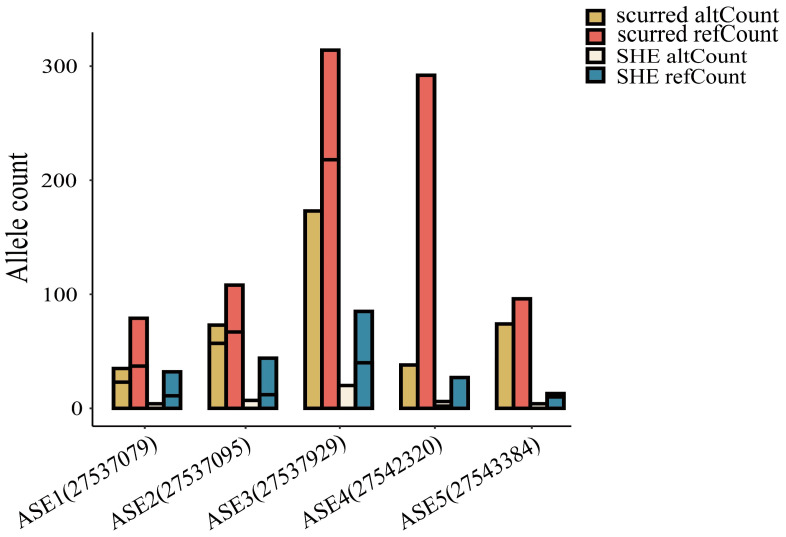
Allele count of five allele-specific expressions (ASEs) that exhibited differences between the SHE (n = 3) and scurred (n = 3) groups.

In Figure 4, five allele-specific expression (ASE) sites were identified: ASE1 (chr11:27537079), ASE2 (chr11:27537095), ASE3 (chr11:27537929), ASE4 (chr11:27542320), and ASE5 (chr11:27543384). These ASE sites were located on exons, with ASE1 and ASE2 on Exon 1, ASE3 on Exon 3, ASE4 on Exon 8, and ASE5 on Exon 14. In the scurred and SHE groups, all sites’ allele counts had the same trend: the alternative counts were lower than the reference counts. Thus, these ASE sites may be strongly associated with the horn phenotype.

### 2.5. Identification of Potential Functional Sites in ALOX12B

**Figure 5:**
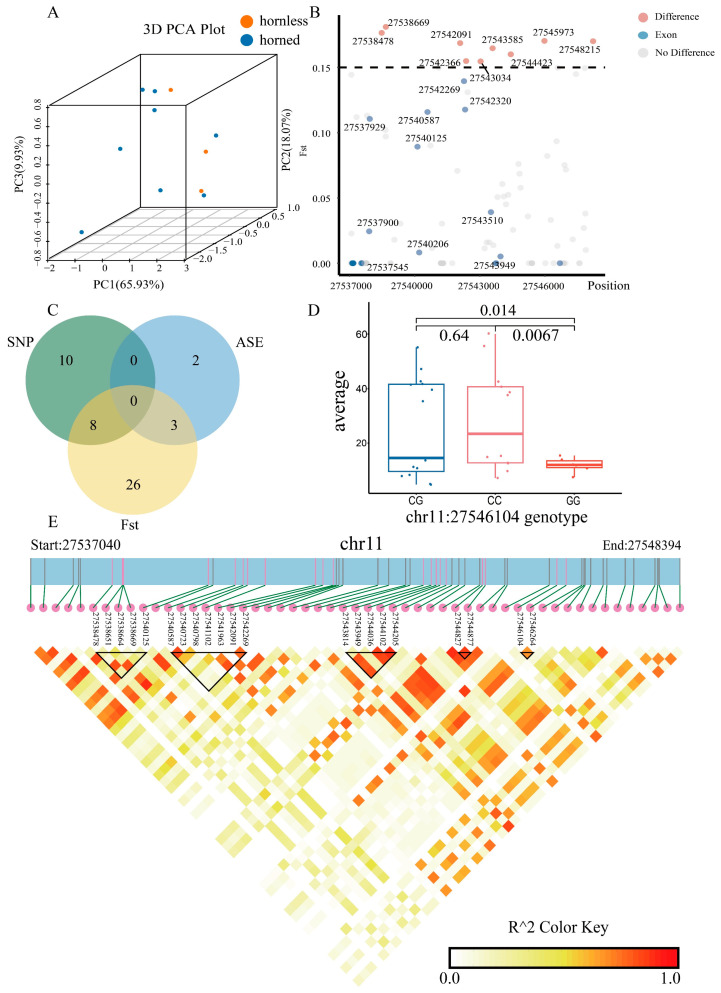
Genetic variation of *ALOX12B*. (**A**) The three-dimensional principal component analysis (3D-PCA) plot of *ALOX12B* using public RNA-seq datasets (n = 2915). Each point on the graph is represented by a sheep breed. According to the breed information, it is divided into horned and hornless types. Yellow represents hornless, and blue represents horned. (**B**) F-statistics (Fst) of functional sites of *ALOX12B* using the public whole genome sequencing (WGS) datasets (n = 3125). (**C**) Intersection of such sites involving single nucleotide polymorphisms (SNPs), ASEs, and Fst > 0.05. (**D**) Boxplot showing the individual horn length for various genotypes (self-tested WGS datasets, n = 34), accompanied by *p*-values calculated through the *t*-test. (**E**) Linkage disequilibrium (LD) heatmap of *ALOX12B* (self-tested WGS datasets, n = 34). LD blocks of SNPs with higher LD scores are represented by black triangles. Red represents a significantly elevated LD score.

In analyzing the 2915 public data, the result of three-dimensional principal component analysis (3D-PCA) showed significant separation between horned and hornless sheep breeds (Figure 5A). This result indicated that *ALOX12B* can, to some extent, distinguish between the two groups, and the sites in this gene region contributed to the regulation of horn development. A total of nine loci with an F-statistic (Fst) > 0.15 were screened by analyzing the public whole genome sequencing (WGS) datasets (n = 3125), and only chr11:27543585 was located on Exon 5 among these loci (Figure 5B, Supplementary Table S3). Through analyzing the self-tested WGS datasets (n = 34), single nucleotide polymorphisms (SNPs, n = 18) were selected. As can be seen in Figure 5C, by intersecting sites with five ASEs and 37 loci (Fst values > 0.05), eight loci were identified (g.27543814G>A, g.27544036T>C, g.27544102G>A, g.27544781A>G, g.27544827T>C, g.27544877C>T, g.27546104C>G, g.27546898T>C), located in the intron region, but their Fst values were not greater than 0.15 (Figure 5C). Among these, chr11:27546104, which had the highest Fst value, showed significant differences in horn length across sheep with different genotypes (Figure 5D). These functional gene loci showed significant differences between horned and hornless sheep and may be crucial in horn size, thereby influencing horn length.

Linkage disequilibrium (LD) revealed 21 SNPs that had high LD scores (Figure 5E). There were two LD blocks containing five SNP loci. LD block1 contained chr11:27538478, chr11:27538651, chr11:27538664, chr11:27538669, and chr11:27540125. LD block3 contained chr11:27543814, chr11:27543949, chr11:27544036, chr11:27544102, and chr11:27544205. LD block2 contained seven SNP sites, which included chr11:27540587, chr11:27540723, chr11:27540798, chr11:27541102, chr11:27541963, chr11:27542091, and chr11:27542269. These findings indicated that these loci were highly likely to affect the growth of horns.

## 3. Discussion

In previous studies, many genes have been reported to be correlated with the growth of horns. However, research using CRISPR/Cas9 technology revealed that although the *RXFP2* gene was partially disrupted, the horn type of sheep remained unaffected [18]. These analyses partially demonstrate that horn development is not merely dictated by a single gene or locus [19].

The transcriptional regulation of *ALOX12B* is still not fully understood. A study showed that during skin barrier formation, 12R-LOX could oxidize O-linoleoyl-ω-hydroxyceramide to 9R-hydroperoxy-linoleoyl-ω-hydroxyceramide; then eLOX3 (epidermal lipoxygenase-3) further converted 9R-hydroperoxy-linoleoyl-ω-hydroxyceramide to epoxyalcohol, promoting the partial hydrolysis of linoleate in O-linoleoyl-ω-hydroxyceramide [20]. This biochemical process allows for the covalent attachment of free β-hydroxyl groups to the proteins of the cornified cell envelope (CE), ultimately contributing to the formation of the corneocyte lipid envelope (CLE) [21]. Together, the CLE and CE constituted the necessary structure of the epidermal water barrier to reduce water loss. The absence of CLE was found in 12R-LOX (null) mice, demonstrating that 12R-LOX could promote the formation of CLE [22]. These studies provide a preliminary basis for understanding *ALOX12B*’s role in skin keratinization. However, to date, research on the role of *ALOX12B* in the growth and development of horns is limited. Although this gene’s mutations are related to skin keratinization, the specific genetic mechanism is unknown.

Our study showed the expression differences of *ALOX12B* between SHE and scurred groups in sheep for the first time and its expression in different sheep breeds and tissues. Our results indicated that compared with the SHE group, the *ALOX12B* was significantly upregulated in the scurred group and expressed at lower levels in the skin. Overall, the most elevated expression levels were recorded in human and sheep skin. It was speculated that the *ALOX12B* gene was strongly associated with the formation of horns, with horn size influenced by its expression level. Overexpression of *ALOX12B* may even inhibit horn growth. However, there is a limitation in our study to analyze sex differences for *ALOX12B* due to the lack of data on male sheep horns. In addition, there were differences between the SHE and scurred groups in the screened SNP sites. However, these results were not verified experimentally. This could potentially compromise the reliability of our findings. Therefore, experimental verification will be performed in a subsequent analysis of this gene. Meanwhile, this study was currently using horn length phenotypic data from Small-tailed Han sheep (n = 34) to consider the heritability of *ALOX12B*. In the future, we will further study *ALOX12B* by combining Tibetan sheep phenotypic studies with genetic analyses, aiming to achieve a more thorough comprehension of the genetic factors that contribute to horn development. This will contribute to a clearer understanding of the relationship between genotype and phenotype in horn development.

Allele-specific expression (ASE) analysis, based on RNA-seq, is widely utilized in pigs [23], cattle [24], and mice [25]. However, there is a relative paucity of ASE research on sheep. This study identified five ASEs, yet no common intersection was observed with the loci (F-statistic values > 0.05) and SNPs. This discrepancy may be attributed to the fact that ASE primarily recognizes cis-trans regulation, whereas SNP primarily identifies mutation sites. Furthermore, the ongoing refinement of genomic information and the analysis of multiple samples will likely provide additional research opportunities in this field, contributing to a deeper comprehension of the mechanisms regulating gene expression in general or specific to horn development.

The neighbor-joining (NJ) method offers a notable advantage in computational efficiency, enabling the rapid construction of evolutionary trees while maintaining high accuracy for extensive datasets. This capability facilitates the effective identification of evolutionary relationships among species. Although NJ may encounter limitations in complex scenarios, such as evolutionary rate heterogeneity and model simplification issues, it was selected for this study’s purpose of constructing an evolutionary tree. The method successfully differentiated between hornless and horned animals. The 15 amino acid sites identified may have significant effects on the evolution of horns.

The elevated expression of *ALOX12B* in the skin and ram testis tissues observed in this study suggested its potential involvement in both physiological and reproductive processes in rams. Given *ALOX12B’*s role in lipid metabolism and inflammatory responses, its expression in the testis may be associated with sperm production, sexual maturation, or seasonal reproductive cycles. Considering the typically seasonal nature of ram fertility, *ALOX12B* could potentially play a role in regulating seasonal variations in reproductive performance. Future research could explore the seasonal fluctuations in *ALOX12B* expression to ascertain whether environmental factors such as light exposure or temperature, known to influence reproductive cycles in various livestock species, affect its activity. Additional experimental validation is necessary to elucidate the specific function of *ALOX12B* in these processes.

*ALOX12B* was also expressed in small amounts in the brain, whereas it was expressed relatively low or almost nonexistent in other tissues (e.g., lipids, heart, large intestine, lungs). This is consistent with our previous findings in pigs, cattle, humans, and sheep. Horn growth and development are tightly linked to skin growth, while ALOX12B protein’s expression in keratinized animals shows a similar trend; this indicates a strong relationship between *ALOX12B* and horn growth.

## 4. Materials and Methods

### 4.1. Ethics Statement

The animal research protocol received approval from the Science Research Department of the Institute of Animal Sciences, Chinese Academy of Agricultural Sciences, Beijing, China (IAS-CAAS), under acceptance number IASCAAS-AE-03.

### 4.2. Animal and Sample Collection

RNA-seq datasets were previously acquired by the research team (PRJNA1003277) [26]. The research team collected the soft horn tissue of Tibetan sheep (female, 3.5 to 5 years old, collected in Dangxiong, Tibet, China). The samples (n = 6) were divided into two groups: the scurred horn group (n = 3), with horn lengths ranging from 0 to 12 cm, and the normal horn group (SHE, n = 3), with horn lengths greater than 12 cm (Supplementary Table S1). All tissues were stored in deep cryopreservation tubes and then immediately frozen using liquid nitrogen.

### 4.3. RNA-Seq Data Analysis

We additionally obtained 2915 publicly available sheep RNA-seq datasets from two databases: the National Center for Biotechnology Information (NCBI) database (https://www.ncbi.nlm.nih.gov/, accessed on 23 July 2023) and the European Bioinformatics Institute (EBI) database (https://www.ebi.ac.uk/, accessed on 23 July 2023). To ensure a robust analysis, a standard workflow was employed to process the data: initially, data were quality controlled and trimmed utilizing Trim Galore (v.0.6.7). Then, sequences were aligned to the reference genome (ARS-UI_Ramb_v2.0) by STAR (v.2.7.7a) [27]. Finally, the clean datasets were generated for in-depth investigation, achieving over 85% unique mapping reads and more than 20,000,000 clean reads. Normalized gene expression levels and exon modeling were performed by calculating the number of transcripts per million exons (TPM) using the Stringtie’s prepDE.py script (v.2.1.5) [28]. The generated data were carried out by featureCounts (v.2.0.1), and the original count matrix was extracted [29]. The GATK (v.4.2.5.0) pipeline, with the default parameters, was applied to Allele-specific expression (ASE) [30,31].

### 4.4. Expression Pattern Analysis of the ALOX12B Gene

We used ggplot2 (v.3.5.1) to generate a boxplot for the self-tested RNA-seq datasets (n = 6). The dexseq_prepare_annotation2.py script (from https://github.com/vivekbhr/Subread_to_DEXSeq, accessed on 5 March 2024) was used to initialize the genome annotation (GTF) file, enabling the analysis of disparities in *ALOX12B* exon expression between the two groups. After that, the formatted GTF file and the counts matrix were processed using the load_SubreadOutput.R. Subsequently, an analysis of differential gene expression in exons was performed.

The public RNA-seq datasets were downloaded from the GTEx project, including pigs (http://piggtex.farmgtex.org/, accessed on 15 August 2023, n = 2651), cattle (https://cgtex.roslin.ed.ac.uk/, accessed on 6 October 2023, n = 4359), and humans (https://gtexportal.org/home/datasets, accessed on 17 January 2024, n = 9810) [32,33,34]. Together with the previous public sheep datasets (n = 2915), all RNA-seq datasets were merged and categorized into 16 tissues. *ALOX12B*’s mean TPM values are shown in Table S2.

The sheep breed information from public RNA-seq datasets (n = 2915) was collected, excluding those with smaller sample sizes, to further explore the specific expression variations of *ALOX12B*. These datasets from ten breeds of sheep were classified into three subgroups: hornless (Spanish churra sheep, Hu sheep, Bashibai sheep, Texel sheep), scurred (Chinese merino sheep, Gansu alpine fine wool sheep, Minxian black fur sheep, Tan sheep), and SHE sheep (Rambouillet sheep, Tibetan sheep). All significance statistics were calculated using the *t*-test.

For the public sheep RNA-seq datasets (n = 2915), principal component analysis (PCA) was conducted by prcomp function in stats (v 4.3.3) with the default parameters, and the three-dimensional (3D) PCA scatterplot was visualized by scatterplot3d (v0.3.44) with the parameter “pch = 19”. The percentage of variance explained for each principal component was PC1 = 65.93%, PC2 = 18.07%, and PC3 = 9.93%.

### 4.5. Cross-Species Comparison of ALOX12B Protein

A total of 16 species’ ALOX12B protein sequence files were retrieved from NCBI, including sheep, goats, camels, dogs, cattle, deer, mice, and humans. Using the MEGA (v 11.0.11), we constructed a protein evolutionary tree for ALOX12B [35]. The ClustalW method (with default parameters) [36,37], the Jones–Taylor–Thornton model, and the neighbor-joining method were applied to align raw data and build an evolutionary tree for the ALOX12B protein. To identify the presence of amino acids in critical positions within ALOX12B, AlphaFold2 (with default parameters) was applied to predict the 3D structure of ALOX12B from UniProt (https://www.uniprot.org/, accessed on 20 June 2024) [38].

### 4.6. Identifying SNP Loci from WGS

The public WGS data were obtained from NCBI for 3125 sheep, including PRJEB30931, PRJNA304478, PRJNA325682, PRJNA479525, PRJNA480684, PRJNA509694, PRJNA624020, PRJNA675420, PRJNA779188, PRJNA783661, and PRJNA822017 [39,40,41,42,43,44,45,46,47,48]. These datasets were classified into two groups: horned and hornless. In order to distinguish SNPs that differed significantly between the two groups, Fst values were calculated by VCFtools (v.0.1.16).

For the experiment, we measured the horn length of 34 Small-tail Han sheep, specifically the average length of the left and right horns, and performed WGS on them. Sequencing reads, and adapters were trimmed by Trimmomatic (v.0.39). Raw sequence quality was determined by using FastQC (v.0.12.1) [49]. After that, BWA (v.0.7.17) and Picard (v. 3.1.1) were used for mapping, sorting, and deduplication [50,51]. Here, the default parameters in the GATK (v.4.2.5.0) pipeline were applied to SNP prediction, while mutation annotation was conducted by SnpEff (v.4.3) [52]. All statistical analyses of significance were performed using a *t*-test. For a more in-depth analysis, the filters “--maf 0.45” and “--min-meanDP 5” in VCFtools (v.0.1.16) [53] were employed, followed by the use of LDBlockShow (v.1.40) [54] to the determination of SNP chains.

## 5. Conclusions

This study showed for the first time that *ALOX12B* expression is related to sheep horn size and type and identified 15 conserved sites in keratinous animals and eight SNPs (Fst > 0.05) associated with horn size in the SHE and scurred horn groups, which may serve as potential molecular markers for genetic selection to reduce horns. Specific amino acid sites (n = 15) that may be significant in the evolutionary development of the ruminant horn were identified in the ALOX12B protein. Additionally, *ALOX12B* was found to be highly expressed in the testis, suggesting a potential role in reproductive traits. These findings provide valuable insights for improving sheep, particularly for controlling horn size and improving reproductive traits.

## Data Availability

For details regarding the RNA-seq datasets, please access the NCBI website using PRJNA1003277.

## Supplementary Materials

The following supporting information can be downloaded at https://www.mdpi.com/article/10.3390/ijms26010079/s1.
